# Does Botulinum Toxin Treatment Affect the Ultrasonographic Characteristics of Post-Stroke Spastic Equinus? A Retrospective Pilot Study

**DOI:** 10.3390/toxins12120797

**Published:** 2020-12-14

**Authors:** Alessandro Picelli, Mirko Filippetti, Camilla Melotti, Flavio Guerrazzi, Angela Modenese, Nicola Smania

**Affiliations:** 1 Department of Neurosciences, Biomedicine and Movement Sciences, University of Verona, 37100 Verona, Italy; mirko.filippetti@univr.it (M.F.); nicola.smania@univr.it (N.S.); 2 Department of Neurosciences, University Hospital of Verona, 37100 Verona, Italy; camilla.melotti@aovr.veneto.it (C.M.); flavio.guerrazzi@aovr.veneto.it (F.G.); angela.modenese@aovr.veneto.it (A.M.)

**Keywords:** achilles tendon, elasticity imaging techniques, muscle spasticity, ultrasonography

## Abstract

Equinovarus/equinus foot is a pattern most commonly treated with botulinum toxin type A in patients with post-stroke spasticity involving the lower limbs; the gastrocnemius is the muscle most frequently injected. Spastic equinovarus/equinus can present a mixture of conditions, including spasticity, muscle/tendon shortening, muscle weakness and imbalance. In this study, we wanted to determine whether botulinum toxin treatment affects the ultrasonographic characteristics of post-stroke spastic equinus. The same dose of AbobotulinumtoxinA was injected into the gastrocnemius medialis and lateralis of 21 chronic stroke patients with spastic equinus. Clinical (Ashworth scale and ankle range of motion) and ultrasound (conventional and sonoelastography) evaluation of the treated leg was carried out before and 4 weeks after injection. No significant effects of botulinum toxin treatment on the ultrasonographic characteristics of spastic equinus were observed. As expected, there were significant improvements in ankle passive dorsiflexion range of motion and calf muscle spasticity at 1 month after treatment. There was a direct association between Achilles tendon elasticity and calf muscle spasticity at baseline evaluation. Larger studies with a long-term timeline of serial evaluations are needed to further investigate the possible effects of botulinum toxin injection on spastic muscle characteristics in patients with post-stroke spasticity.

## 1. Introduction

Botulinum toxin type A (BoNT-A) injection is a first-line treatment for post-stroke spasticity (PSS) [1]. Equinovarus/equinus foot is most commonly treated with BoNT-A in patients with PSS involving the lower limbs; the gastrocnemius is the muscle most frequently injected [2].

Spastic equinovarus/equinus can present with a mix of calf muscle spasticity, tricep surae/Achilles tendon shortening, ankle dorsiflexor weakness and tibialis anterior/peroneus muscle imbalance [3]. This group of conditions may require combined treatment with drugs, physical therapy, orthoses, surgery and other rehabilitation procedures [3,4,5,6].

Increased intramuscular connective tissue and fat content can cause progressive enhancement of muscle echogenicity in post-stroke spastic equinus [7], resulting in gradual loss of response to BoNT-A injection [8]. Muscle structure changes due to PSS can also lead to a progressive decrease in muscle thickness and posterior pennation angle in patients with spastic equinus [7].

Because some “neurological” and “rheological” features of PSS change over time due to chronicity and treatment, with this study we wanted to expand current knowledge in planning the management of lower limb PSS in chronic stroke. To do this, we investigated whether BoNT-A treatment affects the ultrasonographic characteristics of post-stroke spastic equinus.

## 2. Results

For this observational pilot study, we retrospectively evaluated 21 chronic stroke patients with PSS who received BoNT-A injection into the affected gastrocnemius medialis and lateralis between September 2018 and January 2019. Table 1 presents patient demographics and clinical features.

No significant change was noted at the follow-up ultrasonographic evaluation. Clinical evaluation at the follow-up revealed significant changes in ankle passive dorsiflexion range of motion (PROM) (*p* = 0.002), Ashworth scale (*p* = 0.008), Tardieu grade (*p* = 0.008) and Tardieu angle (*p* < 0.001) (Table 2).

Correlation analysis of spastic equinus features at baseline showed a direct association between Achilles tendon hardness percentage and age (*p* = 0.035; ρ = 0.463), and calf muscle spasticity as measured on the Ashworth scale (*p* = 0.045; ρ = 0.441). Time since stroke onset was directly associated with spastic gastrocnemius medialis (*p* = 0.037; ρ = 0.458) and lateralis (*p* = 0.005; ρ = 0.594) echogenicity. There was an inverse correlation between muscle echogenicity of the spastic gastrocnemius lateralis and age (*p* = 0.021; ρ = −0.509) (Table 3).

## 3. Discussion

In this study, we wanted to determine whether botulinum toxin treatment, and not only the progression of PSS over time, can affect the ultrasonographic characteristics of spastic equinus. At the follow-up evaluation (1 month post-injection), we observed no significant effect of BoNT-A injection on ultrasonographic characteristics (i.e., muscle echo intensity, muscle thickness, posterior pennation angle, Achilles tendon thickness and hardness) of post-stroke spastic equinus. This may contrast with current evidence for the effects of BoNT-A on muscular tissue in animal models (development of atrophy and myofibrillar structural changes after injection) and in humans (atrophy and fatty infiltration detected by magnetic resonance imaging 1 year after a single injection into the gastrocnemius lateralis of healthy volunteers; atrophy and reduced pennation angle on ultrasonography at 2 months after injection into the gastrocnemius medialis of stroke patients) [9,10,11]. A plausible explanation for our findings is that the follow-up after injection (only 1 month) may have been too short to reveal possible effects of BoNT-A on the muscle tissue in these patients.

We noted significant improvements in ankle dorsiflexion PROM and calf muscle spasticity after BoNT-A injection into the spastic gastrocnemius medialis and lateralis. These observations are in keeping with the current literature, which reports on the effectiveness of BoNT-A for treating PSS [1].

The Achilles tendon is a key target for the neuro-orthopedic management of spastic equinovarus/equinus [3,4]. Predictably, given that BoNT-A acts on the “neurological” components of PSS, we found no direct or indirect effects of BoNT-A injection into the spastic gastrocnemius medialis and lateralis on the Achilles tendon, which is mainly involved in the spastic “rheological” components of spastic equinovarus/equinus. However, we did note a direct association between Achilles tendon hardness percentage and calf muscle spasticity (as measured with the Ashworth scale) at baseline. The probable reason is that the Ashworth scale evaluates a combination of “neurological” and “rheological” factors related to PSS, in which the increased resistance to movement is due to both stretch reflex activity and increased soft tissue stiffness [12]. From this perspective, our findings, obtained through direct tendon evaluation by sonoelastography, expand on those reported by Freire and colleagues, who observed similar Achilles tendon complacency as measured indirectly by means of the relationship between tendon length and its passive torque in PSS patients and in healthy adults. They concluded that the increased stiffness of the affected ankle was not related to passive Achilles tendon extensibility [13]. In addition, our results suggest that the muscular and the tendinous components both need to be considered when using the Ashworth scale to assess PSS.

The present study has several limitations. The sample size was small and only the gastrocnemius muscle was injected. So, we have no information about the other muscles involved in the spastic equinus/equinovarus pattern, such as the soleus, tibialis posterior, flexor digitorum (longus and brevis), flexor hallucis (longus and brevis), extensor hallucis longus and tibialis anterior [14]. The study did not include a long-term timeline of serial (clinical and ultrasonographic) evaluations after the onset of stroke or BoNT-A treatment, except for follow-up evaluation at 1 month after BoNT-A injection. In addition, we did not evaluate the effects of adjuvant treatments (e.g., physical therapy, casting or shock waves) in combination with BoNT-A on the features of spastic equinus [6]. This was carried out to avert potential bias associated with adjuvant therapies that have been found to act on the ultrasonographic and clinical features of spastic muscles [6,15]. Finally, according to the local regulatory, the dose of AbobotulinumtoxinA injected in adult patients need not to be adjusted for body weight or gender. So, we are unable to provide information about the possible effect of body weight or gender at the same dose of botulinum toxin injection.

Larger prospective studies are needed to overcome the limitations of this study. Future areas of focus are neurophysiological and functional outcomes in a long-term timeline of serial evaluations to investigate the possible effects of BoNT-A injection on spastic muscle characteristics in PSS.

## 4. Materials and Methods

For this single center retrospective (chart review) observational pilot study, we included adult patients (age ≥18 years) with spastic equinus foot (calf muscle tone ≥2 on the Ashworth scale) [12], consequent to chronic stroke (time since onset ≥6 months), both naïve and previously treated with BoNT-A (in the latter case, the time since previous injection was ≥5 months), who received AbobotulinumtoxinA (Ipsen, France) ultrasound-guided injection (dilution 500U/2mL) into the spastic gastrocnemius muscle (250U per head) according to the local regulatory [16]. The need to inject only the gastrocnemius muscle was defined by means of diagnostic nerve block [17]. Exclusion criteria were concurrent involvement in other trials, adjuvant treatment in the 4 weeks following BoNT-A injection [6] or previous treatment with neurolytic/surgical procedures for PSS, and fixed contractures, bone deformities or other neurological/orthopedic conditions involving the affected leg. All subjects gave their written informed consent to participate in the study, which was carried out according to the tenets of the Declaration of Helsinki and approved (6th February 2019; approval code 2066CESC) by the local Ethics Committee (Comitato Etico per la Sperimentazione Clinica delle Province di Verona e Rovigo).

### 4.1. Clinical Evaluation

All patients were evaluated immediately before BoNT-A injection and then again 4 weeks afterwards.

#### 4.1.1. Ultrasonographic Evaluation

**Figure 1:**
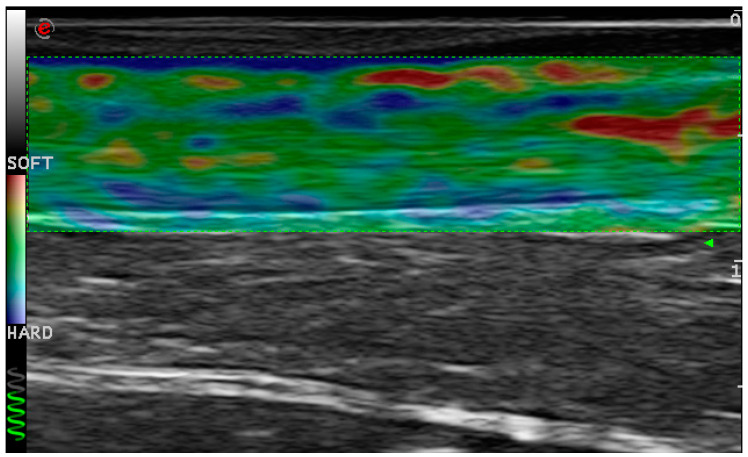
Sonoelastography image of an Achilles tendon.

Ultrasonography with sonoelastography was performed using a MyLab 70 XVision (ESAOTE, Genoa, Italy) device interfaced with a 13 MHz linear probe. The parameters were evaluated at the spastic gastrocnemius medialis and lateralis by conventional ultrasonography: muscle echogenicity graded on the Heckmatt scale (score range from I = normative to IV = very high echo intensity), muscle thickness (distance between the superficial and the deep aponeuroses) and posterior pennation angle (angle between fascicles and superficial aponeurosis) [7,8]. The Achilles tendon of the affected leg was examined 2 to 6 cm proximal to the calcaneal insertion; thickness was measured at the greatest tendon width using conventional ultrasonography; percentage of hardness was evaluated by sonoelastography [17] (Figure 1).

#### 4.1.2. Clinical Evaluation

Affected ankle dorsiflexion PROM was assessed using a goniometer (0° = neutral position) [8]. Calf muscle spasticity was measured using the Ashworth scale and the Tardieu scale. The Ashworth scale is a 5-point scale for grading resistance to rapid passive stretch (0 = no increase in muscle tone; 4 = joint is rigid) [12]. The Tardieu grade measures muscle reaction to fast stretch (0 = no resistance; 1 = slight resistance; 2 = clear catch; 3 = fatigable clonus; 4 = indefatigable clonus). The Tardieu angle is the difference between PROM and the angle of stretch reaction [12].

### 4.2. Statistical Analysis

The data were analyzed using SPSS 26.0 (SPSS Inc., Armonk, NY, USA). Descriptive statistics were calculated. The effects of BoNT-A on ultrasonographic and clinical variables (follow-up vs. baseline) were evaluated using the Wilcoxon test. The correlation between ultrasonographic and clinical variables at baseline was assessed using the Spearman test. Statistical significance was set at *p* < 0.05.

## Figures and Tables

**Table 1:** Demographic and clinical features.

Patients’ Feature	
Age (years) mean (SD)	65.6 (14.5)
Gender (*n*) male/female	15/6
Time since stroke onset (years) mean (SD)	1.7 (0.9)
Stroke etiology (*n*) ischemic/hemorrhagic	16/5
Lesion location (*n*) cortica/subcortica/mixed	4/6/11

Abbreviations: *SD*, standard deviation; *n*, number.

**Table 2:** Raw data and within-group comparisons (Wilcoxon test).

Outcomes	Before Treatment T0	After Treatment T1	T1 vs. T0 *p* Value (95% CI)
Gastrocnemius medialis echo intensity (1–4) median (IQR)	2.0 (1.0; 3.0)	2.0 (1.0; 2.5)	0.705 (−0.32; 0.22)
Gastrocnemius lateralis echo intensity (1–4) median (IQR)	2.0 (1.0; 3.0)	2.0 (1.0; 2.5)	0.739 (−0.35; 0.26)
Gastrocnemius medialis thickness (mm) mean (SD)	14.7 (3.3)	15.4 (3.0)	0.225 (−0.33; 1.51)
Gastrocnemius lateralis thickness (mm) mean (SD)	11.2 (3.1)	11.1 (2.6)	0.823 (−1.33; 0.99)
Gastrocnemius medialis pennation angle (°) mean (SD)	20.8 (7.3)	21.9 (7.2)	0.227 (−0.56; 3.70)
Gastrocnemius lateralis pennation angle (°) mean (SD)	17.4 (5.7)	17.6 (4.9)	0.709 (−2.53; 2.39)
Achilles tendon thickness (mm) mean (SD)	5.1 (0.7)	5.2 (0.6)	0.872 (−0.23; 0.38)
Achilles tendon %HRD mean (SD)	65.9 (12.2)	60.6 (16.4)	0.094 (−12.31; 2.72)
Ankle dorsiflexion PROM (°) mean (SD)	−6.4 (7.3)	−2.2 (5.6)	0.002 (1.57; 7.01) *
Calf muscle spasticity (Ashworth scale, 0–4) median (IQR)	2.0 (2.0; 3.0)	2.0 (1.0; 3.0)	0.008 (−0.87; −0.18) *
Calf muscle spasticity (Tardieu grade, 0–4) median (IQR)	2.0 (1.5; 2.0)	2.0 (1.0; 2.0)	0.008 (−0.55; −0.11) *
Calf muscle spasticity (Tardieu angle, °) mean (SD)	16.9 (5.4)	10.2 (4.3)	<0.001 (−8.87; −4.47) *

Abbreviations: CI, Confidence Interval; IQR, interquartile range; mm, millimeters; SD, standard deviation; °, degrees; %HRD, hardness percentage; PROM, passive range of motion. * Statistically significant (*p* < 0.05).

**Table 3:** Correlation matrix for study variables at baseline (Spearman ρ).

Study Variables	Muscle Echogenicity		Muscle Thickness		Pennation Angle		Achilles Tendon Thickness	Achilles Tendon %HRD
	GM	GL	GM	GL	GM	GL		
Age	0.006	−0.062	0.128	−0.062	−0.173	−0.509 *	0.122	0.463 *
Time since onset	0.458 *	0.594 *	−0.046	−0.089	−0.083	−0.241	−0.127	−0.339
Ankle dorsiflexion PROM	0.076	−0.111	0.011	0.227	−0.185	−0.137	−0.016	−0.171
Calf muscle spasticity								
Ashworth scale	−0.017	0.284	−0.047	0.031	−0.035	0.236	0.134	0.441 *
Tardieu grade	0.238	0.176	−0.387	−0.251	−0.093	0.181	−0.139	−0.048
Tardieu angle	−0.067	0.177	−0.087	0.241	−0.110	−0.051	−0.226	−0.073

Abbreviations: GM, gastrocnemius medialis; GL, gastrocnemius lateralis; %HRD, hardness percentage; PROM, passive range of motion. * Significant correlation (*p* < 0.05).
